# Individual variations and effects of birth facilities on the fecal microbiome of laboratory-bred marmosets (*Callithrix jacchus*) assessed by a longitudinal study

**DOI:** 10.1371/journal.pone.0273702

**Published:** 2022-08-30

**Authors:** Yuko Shigeno, Hong Liu, Chie Sano, Ryo Inoue, Kimie Niimi, Kentaro Nagaoka

**Affiliations:** 1 Laboratory of Veterinary Physiology, Department of Veterinary Medicine, Tokyo University of Agriculture and Technology, Tokyo, Japan; 2 Research Resources Division, RIKEN Center for Brain Science, Saitama, Japan; 3 Laboratory of Animal Science, Department of Applied Biological Sciences, Setsunan University, Osaka, Japan; Sathyabama Institute of Science and Technology, INDIA

## Abstract

Laboratory animals are used for scientific research in various fields. In recent years, there has been a concern that the gut microbiota may differ among laboratory animals, which may yield different results in different laboratories where *in-vivo* experiments are performed. Our knowledge of the gut microbiota of laboratory-reared common marmosets (*Callithrix jacchus*) is limited; thus, in this study, we analyzed the daily changes in fecal microbiome composition, individual variations, and effects of the birth facility in healthy female laboratory-reared marmosets, supplied by three vendors. We showed that the marmoset fecal microbiome varied among animals from the same vendor and among animals from different vendors (birth facility), with daily changes of approximately 37%. The fecal microbiome per vendor is characterized by alpha diversity and specific bacteria, with *Bifidobacterium* for vendor A, *Phascolarctobacterium* for vendor B, and *Megamonas* for vendor C. Furthermore, we found that plasma progesterone concentrations and estrous cycles were not correlated with daily fecal microbiome changes. In contrast, animals with an anovulatory cycle lacked *Megamonas* and *Desulfovibrio* bacteria compared to normal estrous females. This study suggests that the source of the animal, such as breeding and housing facilities, is important for *in-vivo* experiments on the marmoset gut microbiota.

## Introduction

Gut microbiota is now recognized as a multifunctional organ in the body as it plays a role in gastrointestinal health [1], autoimmune diseases [2], and brain function [3–5]. To investigate these complex functions, laboratory animals, especially rodents, have been used in experiments for years. Recently, several studies have indicated that there are differences in the gut microbiota of laboratory animals among different environments, vendors, and strains [6–10]. Since the gut microbiota is associated with other organs, there are concerns that these differences could lead to different results of *in-vivo* experiments [11–13] and poor reproducibility in rodent models [14]; hence, variations in the gut microbiota are now recognized as factors that researchers must consider in *in-vivo* experiments.

The common marmoset (*Callithrix jacchus*) is a tiny new world monkey that originated in northeastern Brazil. Laboratory-bred marmosets have been increasingly used in biomedical research as an alternative non-human primate (NHP) models to macaque monkeys, owing to several advantages including small size, high reproducibility, and ease of handling [15]. Although the use of laboratory marmosets is increasing, the number of studies on gut microbiota composition and the differences in these animal models is limited. Nevertheless, a recent study reported differences in the gut microbiota of laboratory-bred marmosets, which were obtained from different vendors, suggesting that the gut microbiome of marmosets may vary among vendors [16]. Since NHPs, such as marmosets and macaques, have different genetic backgrounds and environments, individual variations in the gut microbiota of common marmosets could be larger than those of inbred rodents in a relatively standardized environment. Individual variations in gut microbiota include daily fluctuation [17] and diurnal variation [18]; thus, a longitudinal study involving sampling a cross-section of marmosets at a specific point in time is essential to evaluate individual variations. Furthermore, because female sex hormones are associated with the gut microbiota in mice and humans [19, 20], they can potentially affect the daily fluctuation and individual variations in marmosets as well. Since female marmosets may develop an anovulatory cycle, the hormonal cycle must be monitored for healthy daily changes.

In this longitudinal study, we analyzed the fecal microbiome of healthy female marmosets to improve our understanding of how the source of the animals can affect the results of in-vivo experiments. This is the first study to highlight the individual variations, including female factors and vendor effects on the fecal microbiome of laboratory marmosets using a longitudinal experimental design.

## Materials and methods

### Ethics statement

This study was conducted in accordance with the Declaration of Helsinki and approved by the Animal Experiments Committee of RIKEN (Saitama, Japan, approval number: H27-2-212, H29-2-211, and W2019-2-011(4)). All animals were cared for and treated humanely in accordance with the institutional guidelines for experiments using animals.

### Animals

Twenty-one female marmosets were born and reared at three vendors, A (the RIKEN Center for Brain Science in Saitama Japan), B, and C. All experiments were conducted at vendor A. Marmosets from vendors B and C were transported to vendor A at least 2 weeks before the experiments begun. The animals were individually housed in stainless steel cages, which were cleaned with water and dried every weekday morning. They were allowed *ad libitum* access to tap water and 40 g/individual/day food pellets (CMS-1M; CLEA Japan Inc., Tokyo, Japan), supplied each day before noon. For animal enrichment, a piece of castella cake was given once a day as a snack at 15:00 h. The animal rooms were controlled at 28 ± 1°C and 50 ± 20% humidity under a 12 h lighting schedule (light from 8:00 to 20:00) and were regularly tested for *Salmonella* spp. and *Shigella* spp. using the culture method.

The animals were weighed periodically. The fecal consistency was monitored, and feces showing a liquid appearance were recorded as diarrhea. Ovulation was confirmed by monitoring the increase in plasma progesterone levels. During the 2-month observation period, 16 marmosets showed a normal estrous cycle, and five were determined to have an anovulatory cycle. Changes in plasma progesterone levels in the anovulatory animals are shown in S1 Fig. Individuals with normal and anovulatory estrous cycles are listed in Table 1 and S1 Table, respectively. Only healthy marmosets were used; individuals that showed body weight loss and diarrhea and exhibited anovulation were excluded.

**Table 1:** List of healthy individuals.

Animal ID	Age (years)	Birth facility	Period of stay in A (month)
Female_1	4.3	A	4.3
Female_2	3.2	A	3.2
Female_3	1.5	A	1.5
Female_4	1.1	A	1.1
Female_5	1.1	A	1.1
Female_6	3.3	B	0.5
Female_7	3.3	B	0.5
Female_8	3.1	B	0.5
Female_9	3.1	B	0.5
Female_10	3.1	B	0.5
Female_11	3.0	B	0.5
Female_12	3.0	B	0.5
Female_13	3.0	B	0.5
Female_14	4.2	C	0.25
Female_15	5.5	C	1.5
Female_16	3.4	C	0.25

### Sample collection

Blood samples (0.3 mL) were collected from the tail or femoral vein using a heparinized syringe under waking conditions every 2–3 days (at 14:00–16:00 h) during the experimental period. Fresh fecal samples were collected (with no urine and sample contamination) in the afternoon from the bottom of the cages using sterile rods and 2.0 mL tubes. Samples were stored at -80°C immediately until DNA extraction.

### Metagenomic analysis of 16S rRNA genes

DNA extraction from fecal samples and purification were conducted using the Kurabo QuickGene DNA tissue kit S (DT-S) (Osaka, Japan) in accordance with the manufacturer’s instructions with a modification of the size of glass beads (0.2 mm diameter). A two-step polymerase chain reaction (PCR) was used for purified DNA samples to obtain sequence libraries. The first PCR was carried out with primer pairs 341F (5′-TCG TCG GCA GC G TCA GAT GTG TAT AAG AGA CAG CCT ACG GGN GGC WGC AG-3′) and 806R (5′-GTC TCG TGG GCT CGG AGA TGT GTA TAA GAG ACA GGG ACT ACH VGG GTA TCT AAT CC-3′) to amplify the V3–V4 region of the 16S rRNA gene. The second PCR was carried out to add the index sequences for the Illumina sequencer with a barcode sequence. The number of amplicons were normalized by SequalPrep Normalization Plate kit (ThermoFisher, Tokyo, Japan) and pooled. Ten pM of the libraries were mixed with phiX control (expected at 20%). The prepared libraries were subjected to sequencing of 285 paired-end bases using the MiSeq Reagent v3 kit on the Illumina MiSeq Next Generation Sequencer (Illumina). The amplicon sequence variant (ASV) table, including quality and chimeric variant filtering, was generated using Quantitative Insights into Microbial Ecology 2 (QIIME2) version 2019.10 with DADA2 plugin [21]. The taxonomy of each ASV was determined by scikit-learn naïve Bayes machine-learning classifier, trained on the Greengenes database version 13.8 (99% OTU dataset). Singletons and ASVs assigned to chloroplast and mitochondria were removed in this study.

### Measurement of plasma progesterone

Blood plasma progesterone was quantified using a competitive enzyme immunoassay (ST AIA-PACK PROGII, Tosoh, Japan) automated by Tosoh AIA System Analyzer (AIA-360, TOSOH) according to the manufacturer’s instructions.

### Statistical analysis

Beta diversity of the fecal microbiome was plotted by principal coordinate analysis (PCoA) on the Bray-Curtis distance and permutational multivariate analysis of variance (PERMANOVA). We assayed for microbial variance using the MicrobiomeAnalyst software with default settings (https://www.microbiomeanalyst.ca). A dendrogram was constructed with the Ward method using the Bray–Curtis distance. The fecal microbial similarity was calculated based on the Bray–Curtis distance, with R software version 3.6.2 using the “vegan” package (version 2.5–6, https://CRAN.R-project.org/package=vegan). The comparison of the fecal microbiome similarity was conducted using the Kruskal–Wallis test followed by the Duhn’s test or Mann–Whitney test using GraphPad Prism software version 8.4.3. A linear discriminant analysis effect size (LEfSe) analysis was performed to discriminate microbial features between groups using MicrobiomeAnalyst. Alpha-diversity and relative abundance of microbial features were compared using the Kruskal–Wallis test followed by Duhn’s test using GraphPad Prism. The receiver operating characteristic (ROC) curves were drawn for specific variables (genera) and the area under the ROC curve (AUC) using GraphPad Prism. This analysis was conducted after a centered log-ratio transformation of microbiome data, with R using the “propr” package (version 4.6.2, https://cran.r-project.org/web/packages/propr/index.html). The models were compared with goodness-of-fit tests, including the corrected Akaike information criterion. For correlation analysis between the relative abundance of genera and plasma progesterone concentrations, Spearman rank correlation coefficient was calculated using GraphPad Prism.

## Results

### Fecal microbiome variation in healthy female marmosets affected by individual variation and vendors

In the metagenomic analysis of 16S rRNA genes, according to the rarefaction curve, only samples with over 15,000 reads were used for the following analyses. A total of 12,343,552 reads were obtained (average: 32,555 reads/samples; range: 15,493–55,711 reads) from 381 samples. From these data, 3,615 amplicon sequence variants (ASVs) and 223 genera were identified. Stacked area plots for the relative abundance of the genera in the individual animal are shown in S2 Fig. Furthermore, 14 genera appeared multiple times in the gut microbiome of all individuals with a relative abundance of 1% or greater during the experiment. The relative amounts of these bacteria in 16 healthy female marmosets are shown in S2 Table.

**Fig 1:**
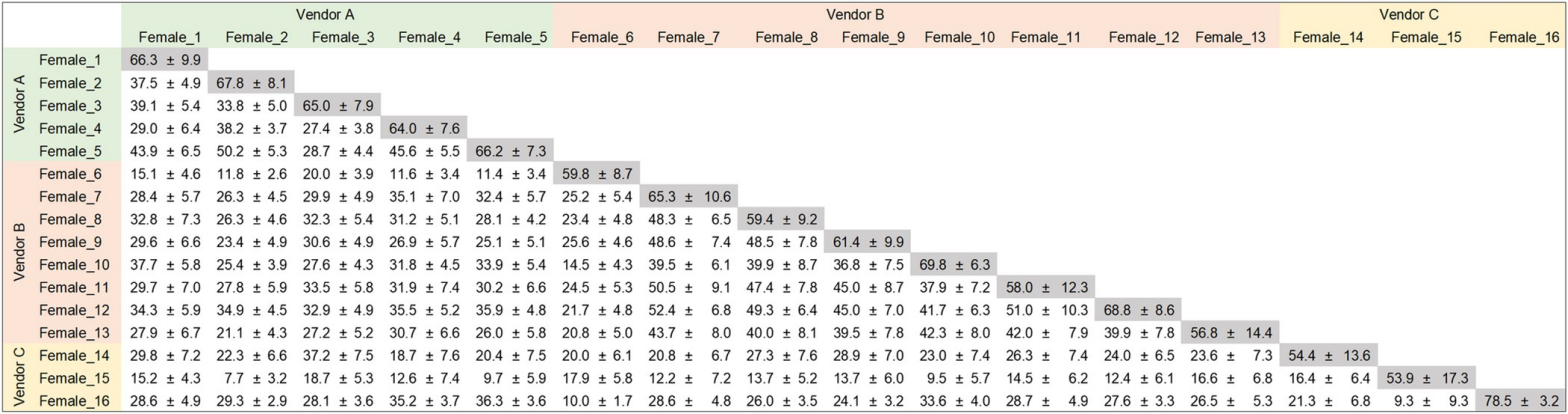
Intra-individual (daily fluctuation) and inter-individual (Individual variation) fecal microbiome similarity among individuals. Values (%) are expressed as mean ± SD. Intra-individual fecal microbiome similarities are filled with gray color.

To understand the daily changes in the fecal microbiome of female marmosets, we calculated intra-individual fecal microbiome similarities in ASV levels in 16 animals during the 2-month observation period (Fig 1, gray color cells). The average for all animals was 62.79 ± 11.17% (mean ± SD), indicating that approximately 37% of the fecal microbiome changed daily within the observation period. The intra-individual similarity was not affected by the vendor and age of the animal (S3 Fig). We also investigated individual variation by calculating inter-individual fecal microbiome similarities in ASV levels among the 16 marmosets (Fig 1, white color cells). The inter-individual similarities among animals from different vendors were lower than those among animals from the same vendor (Fig 1 and S3 Table).

**Fig 2:**
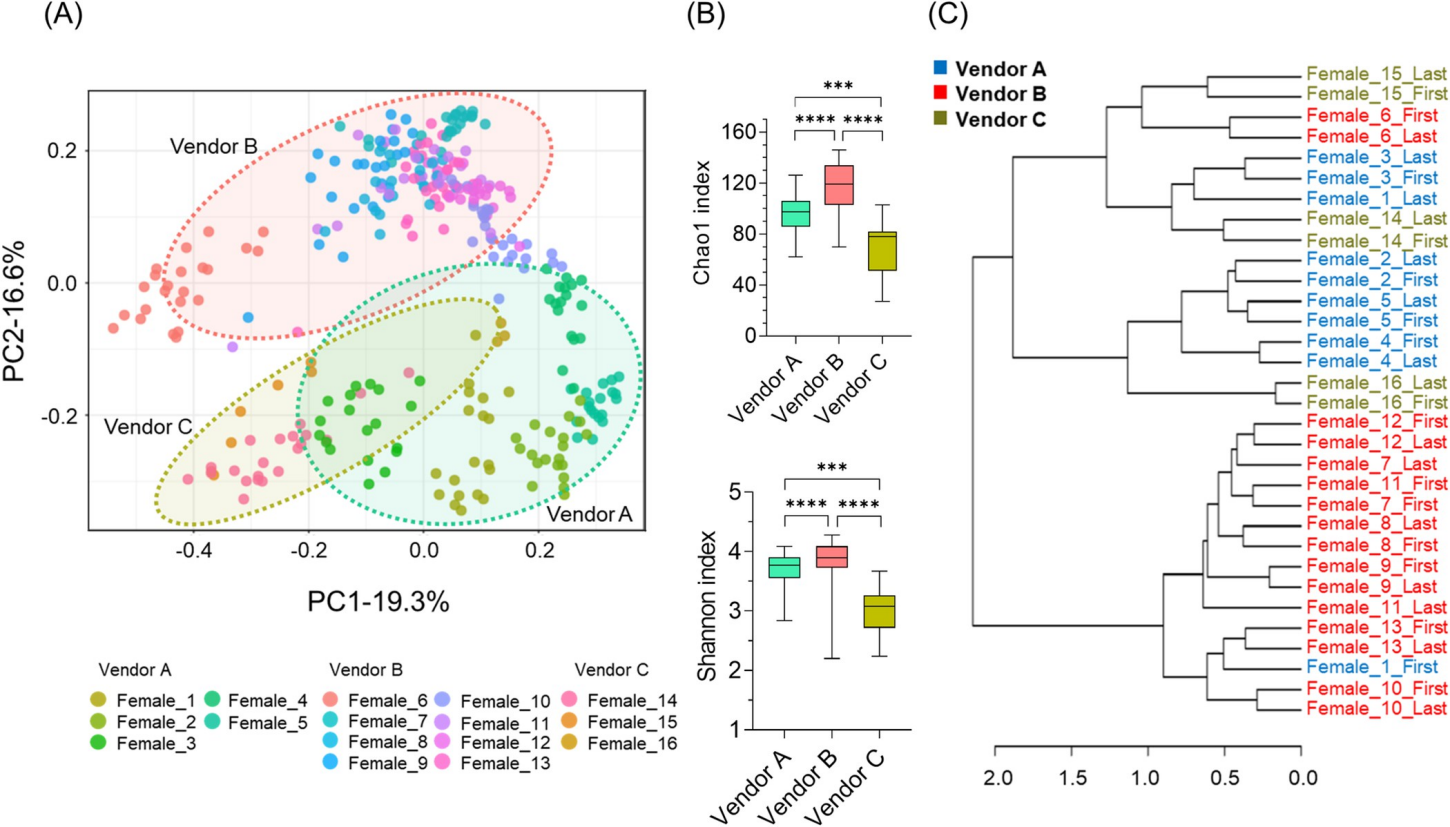
Fecal microbiome differences among vendors. (A) PCoA plot based on the Bray-Curtis dissimilarity index using ASV data of 316 samples from healthy marmosets. The points are colored by animal ID, and each eclipse shows each vendor. The PERMANOVA test was conducted when grouped by individuals and vendors. F-value: 48.886, 44.511; R-squared: 0.70967, 0.22144; both had a p-value of <0.001. (B) Box plots showing a comparison of alpha diversity calculated based on Chao1 and Shannon indices among vendors. The box extends from the 25th to 75th percentiles, and the line of middle of the box shows the median. The ends of the whiskers represent the smallest and largest values. The Kruskal-Wallis test was conducted for each comparison; p-value: ****, <0.0001. ***, <0.001. (C) Dendrogram constructed with first and last fecal microbiome over experimental period by ward methods, based on the Bray-Curtis dissimilarity index.

As shown in Fig 2A, we visualized the individual variations with a PCoA plot and showed significant grouping by vendor (Fig 2A). The differences in fecal microbiome diversities, Chao1 (richness), and Shannon (evenness) indices were also observed among the vendors (Fig 2B). In addition, a dendrogram analysis using samples from the first and last day of the 2-month observation period showed that animals supplied by vendors A and C had similar fecal microbiome, whereas animals supplied by vendor B had different microbiomes (Fig 2C), suggesting that individuals supplied by vendor B could retain the original fecal microbiome after transport to vendor A without major changes.

### Bacterial characteristics of the fecal microbiome of marmosets from different vendors

**Fig 3:**
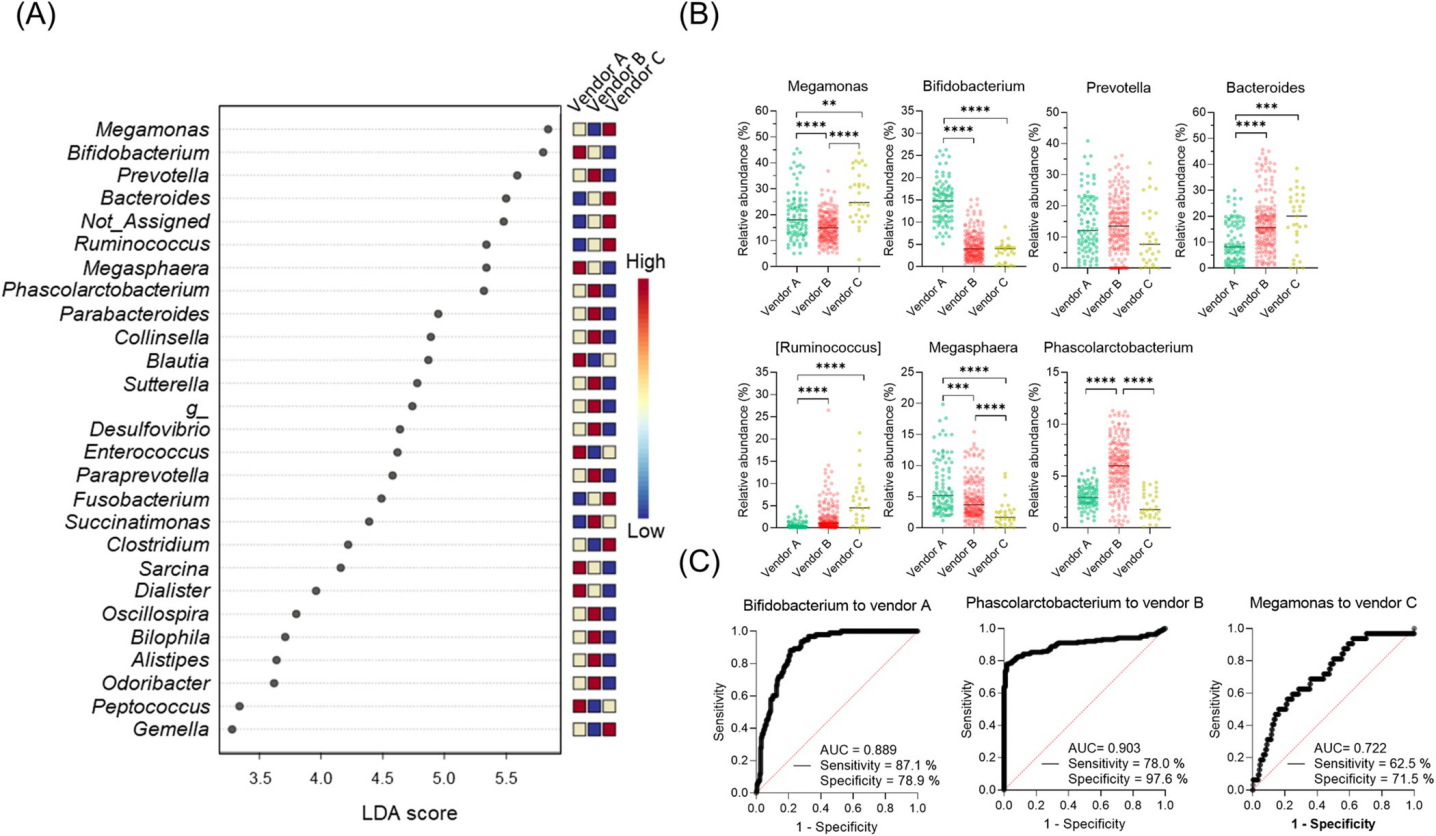
Bacterial characteristics among the fecal microbiome of vendors. (A) Results of the LEfSe analysis comparing vendors. FDR-adjusted p-value <0.05; the LDA log score is 2.0; figures showed an LDA score over 3.0 as the absolute value. Color bar scale shows the LDA score height. (B) Scatter plots comparing the relative abundance of seven genera among vendors. Each symbol shows each sample, and the line of middle of the box shows the median. Kruskall-Walis test followed by Dunn’s multiple comparison test; p-value: ****, <0.0001. ***, <0.001. **, <0.01. (C) ROC curves with the bacterial genera characteristic of each vendor.

To discriminate between the bacterial characteristics of marmosets obtained from the three vendors, we conducted an LEfSe at the genus level and selected seven genera. Based on the linear discriminant analysis (LDA) score, animals from vendor A were characterized by *Bifidobacterium* and *Megasphaera*, animals from vendor B were characterized by *Phascolarctobacterium* and *Prevotella*, and animals from vendor C were characterized by *Megamonas*, *Bacteroides*, and *Ruminococcus* (Fig 3A and 3B). To further demonstrate the association of these genera with the animals from each vendor, we performed a ROC curve analysis (S4 Fig). A comparison of AUC values revealed that three genera were identified as specific bacteria in each vendor (Fig 3C). Using a cutoff value of 8.66% as the relative abundance of *Bifidobacterium*, it is possible to characterize animals from vendor A with 87.1% sensitivity and 78.9% specificity and animals from vendor B with 78.0% sensitivity and 97.6% specificity at a cutoff value of 4.02% for *Phascolarctobacterium* and animals from vendor C with 62.5% sensitivity and 71.5% specificity at a cutoff value of 14.43% for *Megamonas*.

### Estrous cycle and fecal microbiome

To confirm whether the estrous cycle in females affects the daily changes in the fecal microbiome, we searched for bacteria that correlated with blood progesterone concentrations at the genus level using the Spearman correlation analysis (S4 Table). Our results revealed that *Lactobacillus* showed the highest correlation coefficient (r = 0.19); however, correlation coefficients of <0.2 were judged as indicating almost no correlation, suggesting that the estrous cycle had little effect on daily changes in the fecal microbiome in female marmosets.

**Fig 4:**
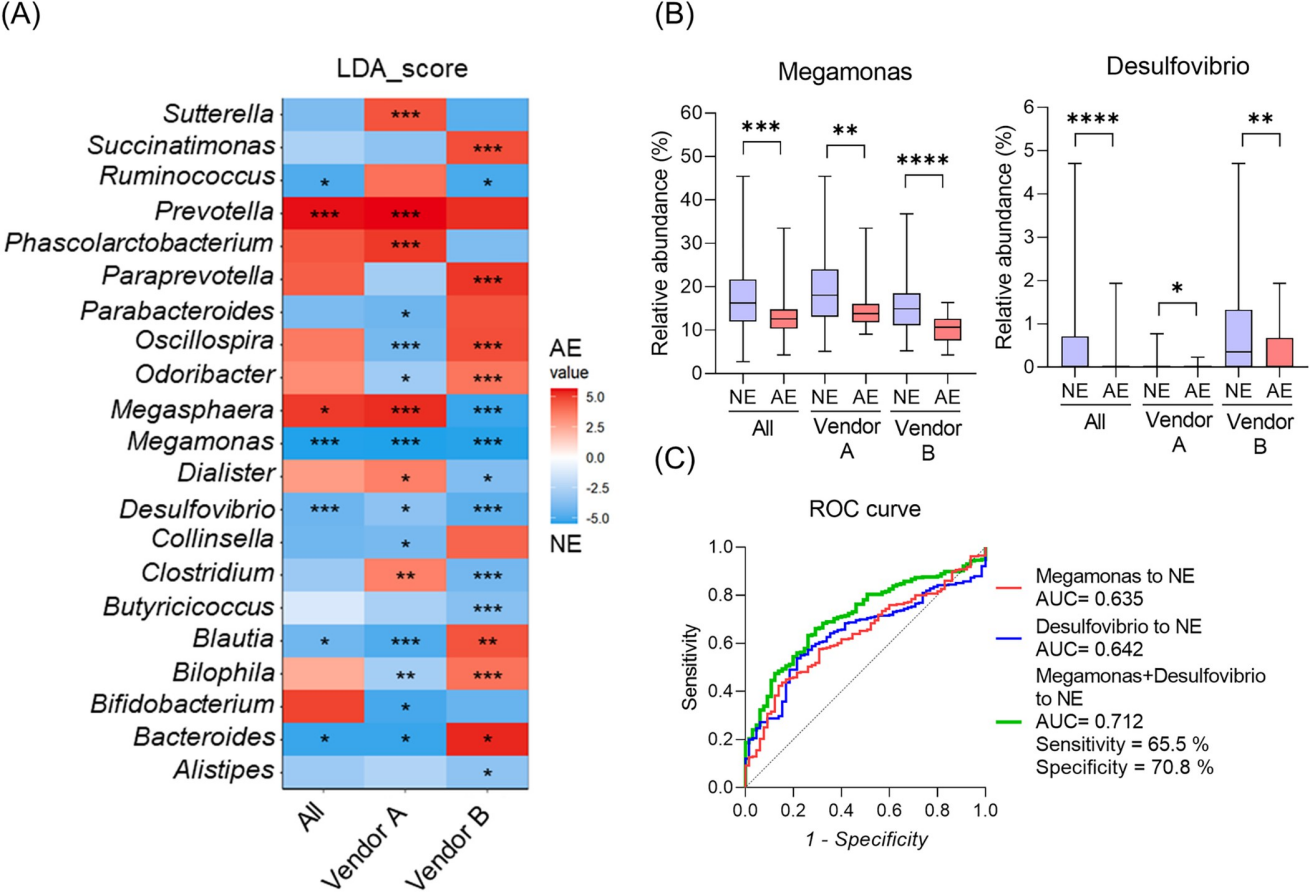
Comparison of fecal microbiome between normal and anovulatory estrous groups. (A) Results of the LEfSe comparison between normal estrous group (NE) and anovulatory estrous group (AE) divided by vendors or individual marmosets. Color bar scale shows LDA sore; red means AE abundance, and blue means NE abundance; p-value: ***, <0.001 **, <0.01, *, <0.05. (B) Box plots showing a comparison of the relative abundance of *Megamonas* and *Desulfovibrio* between NE and AE. The box extends from the 25th to 75th percentiles, and the line in the middle of the box shows the median. The ends of the whiskers represent the smallest and largest values. Kruskal-Wallis test, p-value: ****, <0.0001, ***, <0.001, **, <0.01, *, <0.05. (C) ROC curve analysis using *Megamonas*, *Desulfovibrio*, and a combination of *Megamonas* and *Desulfovibrio*.

As mentioned above, there were five female marmosets (three supplied by vendor A and two by vendor B) that showed an anovulatory estrous cycle during this experimental period, and they were excluded from the previous analyses. Therefore, we performed an LEfSe analysis to investigate the differences in the fecal microbiome between animals with normal and anovulatory estrous cycles and found high LDA scores and significant differences in several bacteria (Fig 4A). Among them, *Megamonas* and *Desulfovibrio* were found to be more abundant in normal animals from all vendors (Fig 4A). A comparison of the relative abundance of *Megamonas* and *Desulfovibrio* is shown in Fig 4B. Furthermore, the ROC curve analysis suggested that it is possible to characterize normal animals with 65.5% sensitivity and 70.8% specificity by using a combination of *Megamonas* and *Desulfovibrio* (Fig 4C).

## Discussion

In this study, regular observation of the fecal microbiome of 16 healthy female marmosets, sourced from three different vendors, over a period of two months showed that individual variations in the fecal microbiome of laboratory-reared female marmosets were greater than the daily changes and that the birth facility (vendor) of the animals also affected the gut microbiota. In addition, the presence of characteristic bacteria in each supplied vendor suggests that it is possible to trace the source of the animals. In contrast, there was no correlation between daily changes in the fecal microbiome and changes in blood progesterone concentration (indicator of the sexual cycle); however, there were differences in the fecal microbiome between normal and anovulatory estrous cycling animals.

The gut microbiome of humans undergoes diurnal variation; in addition, diurnal variation was observed in approximately 15% of OTUs in mice [17, 18, 22, 23]. Therefore, in this study, sampling was performed at a fixed time to avoid the effects of diurnal variation. We showed that the individual variations were greater than the daily changes. In a previous study, the individual variations were greater in the fecal microbiome of marmosets than in that of humans, under uncontrolled diet conditions [24]. Notably, the fecal microbiome of marmosets showed greater individual variations than that of other animal species, even when fed the same diet in a controlled environment [25]. Future studies are needed to investigate the hidden factor generating the fecal microbiome variation among individuals.

The fecal microbiome, including alpha diversity and constituent bacterial species, differed based on the supplied vendors, even in the same environment. This result agrees with a previous study by Sheh et al. [16]. They suggested that the basic composition of the gut microbiota of captive marmoset may be influenced by the breeding facility (supplied vendor) rather than sex or age. In mice, longitudinal studies have shown differences in the alpha diversity and fecal microbiome composition between two suppliers from 3 to 24 weeks of age [26], and Ma et al. reported that in response to changing the cages in which the mice were housed, the fecal microbiota changed within one day but returned to normal after five days of acclimation [27]. This resilience of the gut microbiota has also been reported in humans during travel [28]. Although we did not analyze the fecal microbiome before transport in this study, previous studies indicate that the effects of transport do not appear to last for a long time. Our results suggest that the basic composition of the gut microbiota established at the birth facility in marmosets is maintained after the transfer to other facilities, suggesting the possibility of identifying the birth facility of an animal by identifying the bacterial characteristics of the animals from a specific vendor. Therefore, further research on the large influence of gut bacteria using marmosets is necessary, conducted by grouping the animals according to their birth facility.

For the animals used in this study, *Bifidobacterium*, *Phascolarctobacterium*, and *Megamonas* were found to be characteristic bacterial genera in vendors A, B, and C, respectively. *Bifidobacterium* is widespread in common marmosets [29–32] and is thought to contribute to the unique ability of marmosets to subsist on host-indigestible carbohydrates, including milk oligosaccharide [33, 34]. The abundance of *Bifidobacterium* in the gut microbiome increased during the process of establishing marmoset colonies without specific pathogens, including bacteria, viruses, and parasites [35]. In addition, *Bifidobacterium* was also reported to affect a variety of physiological functions, including those of the brain [36, 37]. *Phascolarctobacterium*, initially isolated from koalas, is widespread in humans, primates, and marmosets and requires succinate for growth and produces propionate [38, 39]. In humans, *Phascolarctobacterium* was associated with the suppression of obesity [40]. *Megamonas* was also reported to be related to obesity and Crohn’s disease patients in humans [41, 42]. In marmosets, *Megamonas* was abundant in IBD progressors and duodenal strictures [16, 43]. Future detailed studies of the physiological characteristics of the animals from each vendor will reveal the biological significance of the bacterial genera that characterize animals from these vendors and their potential involvement as experimental animals in the reproducibility of in-vivo studies.

In this study, *Megamonas*, *Bacteroides*, and *Prevotella* showed relatively high abundance ratios in the fecal microbiome of marmosets and were the top 3 of 14 genera found in the fecal microbiomes shared by 16 female marmosets. Sheh et al. reported that *Bacteroides* and *Prevotella* are the major enteric bacterial species of marmosets and characterized source-associated microbiomes [16]. In this study, the LEfSe results showed *Prevotella*, which characterized vendor B, but the relative abundance comparison and the ROC curve result were not significantly different. The frequency of *Prevotella* is known to be high in chimpanzees, and the *Prevotella*-dominant type has been reported to be associated with a high-carbohydrate diet in humans; therefore, the high prevalence of *Prevotella* in chimpanzees may be due to the consumption of carbohydrate-rich fruits [44]. In this study, experimental marmosets were fed castella cake as a snack after procedures such as blood sampling, and being rich in carbohydrates, it may have caused the high abundance of *Prevotella*.

The association of sex hormones with the composition of gut microbiota has been thoroughly studied in mice; gonadectomy changed the composition of the gut microbiota [45], indicating an effect of sex hormones. In addition, the gut microbiota undergoes sex-specific changes during puberty that influence hormone levels [46]. In female marmosets, *Lactobacillus* was detected as a bacterial genus correlated with changes in plasma progesterone concentration, consistent with a previous study on mice [19]; however, the correlation coefficient was low, at 0.19. In a cross-sectional study using marmosets, the fecal microbiome of marmosets was reported to be unaffected by sex [16]. Collectively, these results suggest that sex hormones have little to no effect on the fecal microbiota of experimental marmosets. In contrast, two genera, *Megamonas* and *Desulfovibrio*, were abundant in marmosets showing a normal estrous cycle. Although we were unable to determine whether the changes in the abundance of these genera are the cause or consequence of the abnormal estrous cycle (anovulation), monitoring the fecal microbiota of female marmosets may allow for the management of the estrous cycle. In addition to anovulatory estrous cycles, other biological conditions may also influence the marmoset fecal microbiota.

In this study, we propose that the fecal microbiota of healthy female marmosets is affected by individual variation, source of the animals, and laboratory environments. Furthermore, this study highlighted the importance of the background (source) of laboratory marmosets, which may influence in-vivo experiments by affecting the gut microbiota.

## Supporting information

S1 Fig Plasma progesterone concentration of anovulatory female marmosets. The graph with red frame shows normal movement of plasma progesterone concentration. The concentration of progesterone detecting ovulation is shown as broken line at 10 ng/mL.
(TIF)

S2 Fig Stacked area plots by each individual based on genus level. Each chart shows the stacked area plot of the gut microbiome of each female over the experimental period.
(TIF)

S3 Fig Intra-individual (daily fluctuation) fecal microbiome similarity (%) based on ASV level. (A) Comparison among animals supplied from three vendors by Kruskall-Walis test. (B) Comparison among animals grouped at age by Mann-Whitney test. The black lines show means, with the dot showing each animal’s intra-individual fecal microbiome similarity.
(TIF)

S4 Fig ROC curve shows the single genus model and genera combination model. *Bifidobacterium* and *Megasphaera* predict vendor A. *Phascolarctobacterium* and *Prevotella* predict vendor B. *Megamonas*, *Bacteroides* and *Ruminococcus* predict vendor C.
(TIF)

S1 Table List of anovulatory individuals. (PDF)

S2 Table Relative abundance and prevalence of bacterial features shared in healthy female marmosets. (PDF)

S3 Table Average similarity of inter-individual (individual variation) fecal microbiome among vendors. (PDF)

S4 Table Genera correlated with plasma progesterone concentration. (PDF)
