# Supplementary figures and images for: Individual variations and effects of birth facilities on the fecal microbiome of laboratory-bred marmosets (Callithrix jacchus) assessed by a longitudinal study
Source: PLoS One. 2022 Aug 30;17(8):e0273702. doi: 10.1371/journal.pone.0273702 (PMC9426884; doi:10.1371/journal.pone.0273702)

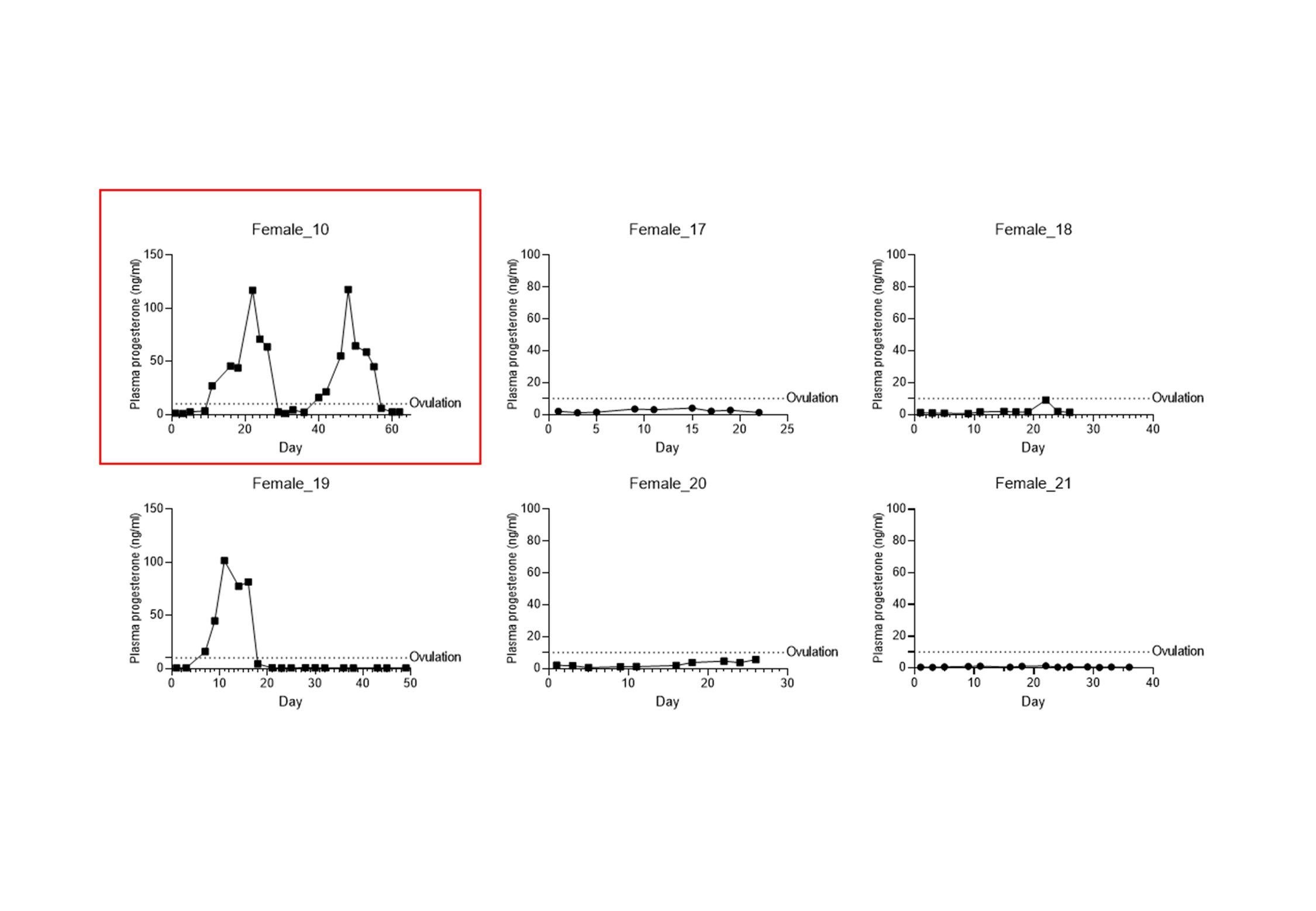

Supplement: S1 Fig — The graph with red frame shows normal movement of plasma progesterone concentration. The concentration of progesterone detecting ovulation is shown as broken line at 10 ng/mL. (TIF) [file pone.0273702.s001.tif]

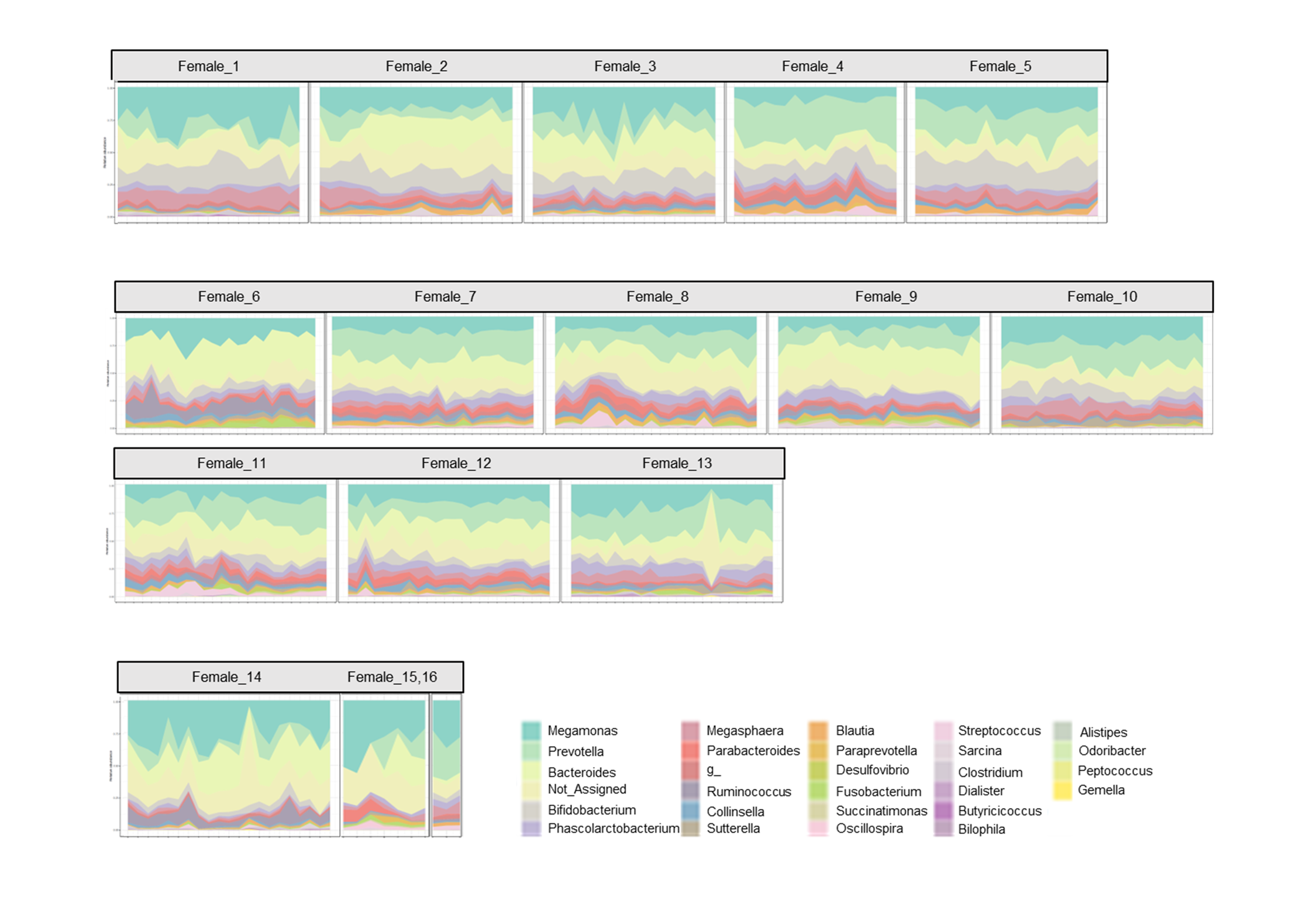

Supplement: S2 Fig — Each chart shows the stacked area plot of the gut microbiome of each female over the experimental period. (TIF) [file pone.0273702.s002.tif]

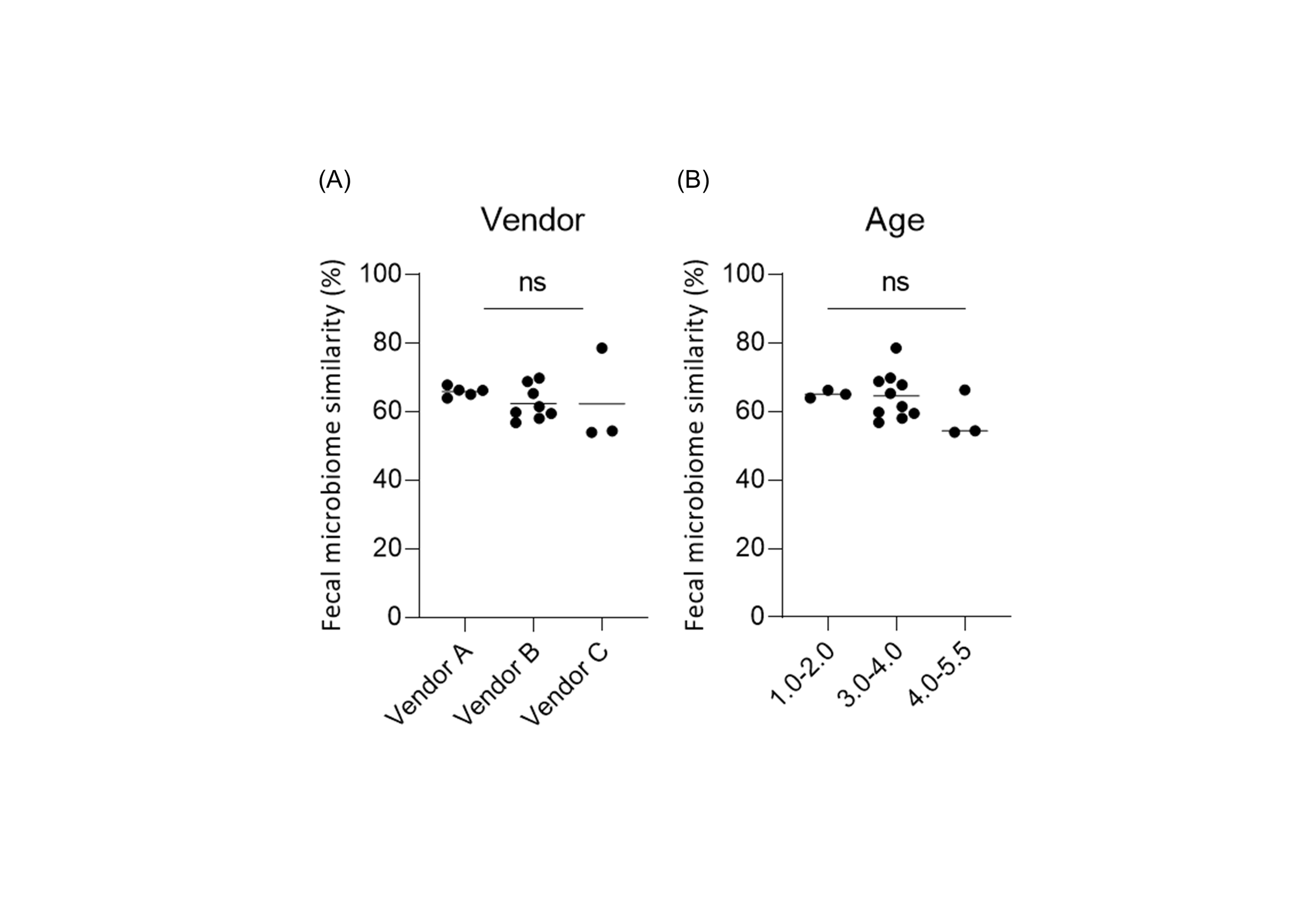

Supplement: S3 Fig — (A) Comparison among animals supplied from three vendors by Kruskall-Walis test. (B) Comparison among animals grouped at age by Mann-Whitney test. The black lines show means, with the dot showing each animal’s intra-individual fecal microbiome similarity. (TIF) [file pone.0273702.s003.tif]

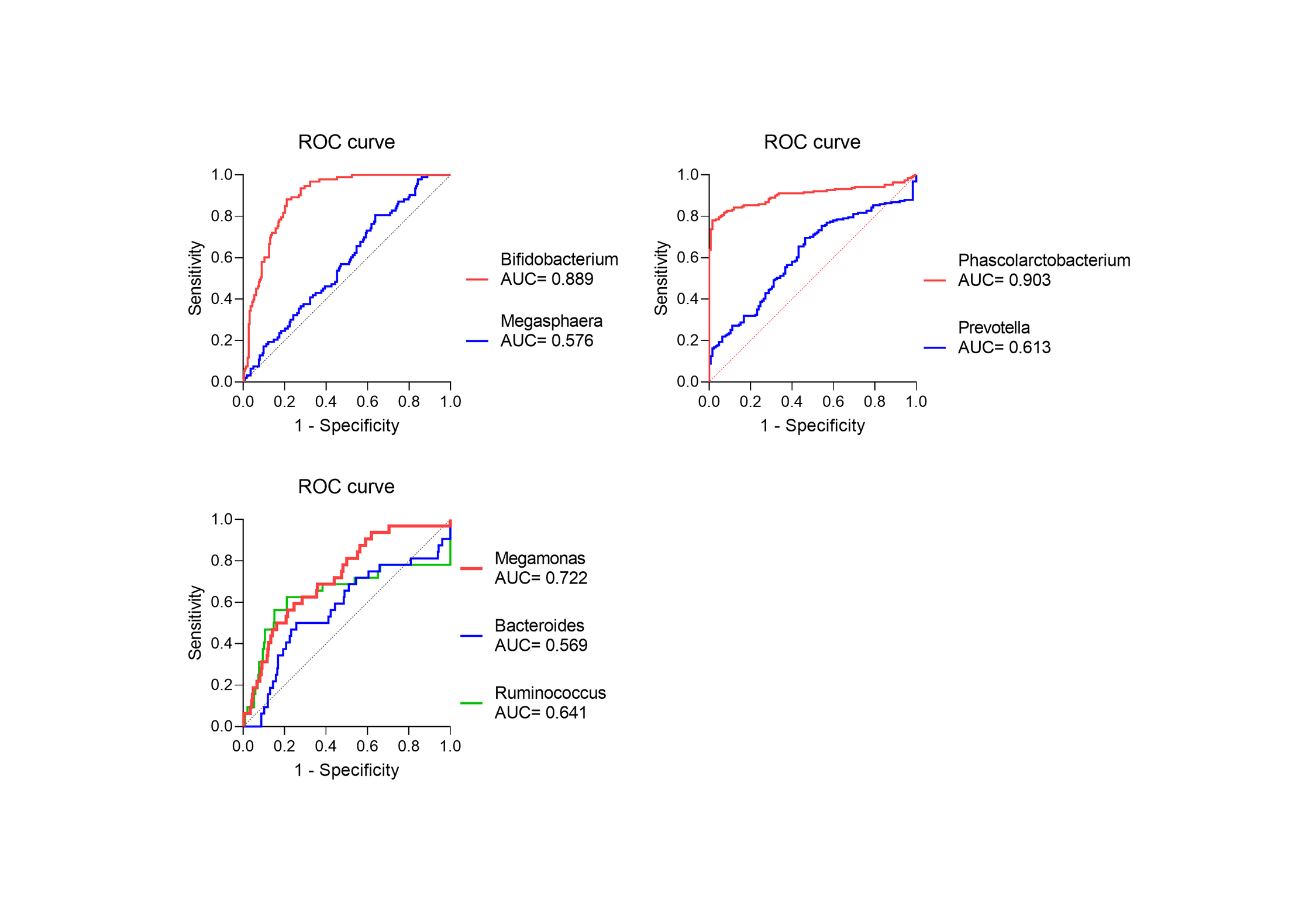

Supplement: S4 Fig — Bifidobacterium and Megasphaera predict vendor A. Phascolarctobacterium and Prevotella predict vendor B. Megamonas, Bacteroides and Ruminococcus predict vendor C. (TIF) [file pone.0273702.s004.tif]
