# Supplementary material for: Individual variations and effects of birth facilities on the fecal microbiome of laboratory-bred marmosets (Callithrix jacchus) assessed by a longitudinal study
Source: PLoS One. 2022 Aug 30;17(8):e0273702. doi: 10.1371/journal.pone.0273702 (PMC9426884; doi:10.1371/journal.pone.0273702)
Supplement: S1 Table — (PDF) [file pone.0273702.s005.pdf]

S1 Table. The list of anovulatory individuals

Animal ID	Age (year)	Birth facility	Period of stay in A(month)
Female_17	4.3	A	4.3
Female_18	1.1	A	1.1
Female_19	1.5	A	1.5
Female_20	3.2	B	0.5
Female_21	3.1	B	0.5
