# Supplementary material for: Individual variations and effects of birth facilities on the fecal microbiome of laboratory-bred marmosets (Callithrix jacchus) assessed by a longitudinal study
Source: PLoS One. 2022 Aug 30;17(8):e0273702. doi: 10.1371/journal.pone.0273702 (PMC9426884; doi:10.1371/journal.pone.0273702)
Supplement: S2 Table — (PDF) [file pone.0273702.s006.pdf]

S2 Table. Relative abundance of bacterial features shared in marmosets

Taxonomy	Relative abundance (%)		
Megamonas	17.72	±	8.15
Bacteroides	15.29	±	10.45
Prevotella	13.52	±	9.10
Bifidobacterium	7.55	±	5.89
Fusobacteriaceae;Other	6.39	±	5.72
Megasphaera	4.97	±	3.69
Phascolarctobacterium	4.68	±	2.60
[Prevotella]	3.41	±	3.47
Parabacteroides	2.82	±	2.94
Collinsella	2.02	±	1.39
Lachnospiraceae;Other	1.54	±	1.03
Sutterella	1.37	±	1.41
Bifidobacteriaceae;Other	1.31	±	1.21
Blautia	1.23	±	1.23

Only relative abundance >1.0 % are shown.
