# Supplementary material for: Individual variations and effects of birth facilities on the fecal microbiome of laboratory-bred marmosets (Callithrix jacchus) assessed by a longitudinal study
Source: PLoS One. 2022 Aug 30;17(8):e0273702. doi: 10.1371/journal.pone.0273702 (PMC9426884; doi:10.1371/journal.pone.0273702)
Supplement: S3 Table — (PDF) [file pone.0273702.s007.pdf]

S3 Table. Average of inter-individual (Individual variation) fecal microbiome similarity among vendors

Vendor	A	B	C
A	37.49 ± 8.94		
B	28.11 ± 8.37	38.92 ± 12.62	
C	23.39 ± 10.77	22.02 ± 8.61	17.12 ± 7.21

Values (%) are expressed as mean ± SD.
