# Supplementary material for: Individual variations and effects of birth facilities on the fecal microbiome of laboratory-bred marmosets (Callithrix jacchus) assessed by a longitudinal study
Source: PLoS One. 2022 Aug 30;17(8):e0273702. doi: 10.1371/journal.pone.0273702 (PMC9426884; doi:10.1371/journal.pone.0273702)
Supplement: S4 Table — (PDF) [file pone.0273702.s008.pdf]

S4 Table. The genera correlated with plasma progesterone concentration

ALL	Spearman's r	P value
Paraprevotella	-0.15	**
Alistipes	-0.15	**
OD1;c__;o__;f__;g__	-0.14	*
mitochondria;g__	-0.14	*
Alcaligenaceae;g__	-0.13	*
[Barnesiellaceae];Other	-0.13	*
Macrococcus	-0.13	*
Erysipelotrichaceae;Other	-0.12	*
Veillonellaceae;g__	-0.12	*
Gammaproteobacteria;Other;Other;Other	-0.11	*
Eubacterium	-0.11	*
Erysipelotrichaceae;g__	-0.11	*
Proteus	-0.11	*
Erysipelotrichaceae;g__Clostridium	0.11	*
Clostridiaceae;g__Clostridium	0.11	*
RFN20	0.11	*
Helicobacteraceae;Other	0.12	*
Butyricicoccus	0.12	*
Kocuria	0.14	*
Rhizobium	0.16	**
Lactobacillus	0.19	***

p value, ***, $p < 0.001$. **, $p < 0.01$. *, $p < 0.05$.
